# Apolipoprotein A1 is associated with SYNTAX score in patients with a non-ST segment elevation myocardial infarction

**DOI:** 10.1186/s12944-019-1101-9

**Published:** 2019-08-07

**Authors:** Bang-Dang Chen, Xiao-Cui Chen, Yi-Ning Yang, Xiao-Ming Gao, Xiang Ma, Ying Huang, Xiao-Mei Li, Min-Tao Gai, Fen Liu, Shuo Pan, Yi-Tong Ma

**Affiliations:** 1 Clinical Medical Research Institute of First Affiliated Hospital of Xinjiang Medical University, Urumqi, China; 2 State Key Laboratory of Pathogenesis, Prevention and Treatment of High Incidence Diseases in Central Asia, Urumqi, China; 3 Xinjiang Key Laboratory of Cardiovascular Research, Urumqi, Xinjiang China; 4 Department of Cardiology, First Affiliated Hospital of Xinjiang Medical University, Urumqi, China; 5 First Department of Cardiology, People’s Hospital of Shaanxi Province, Xi’an, China

**Keywords:** Apolipoprotein A1, SYNTAX score, NSTEMI, Lipid

## Abstract

**Background:**

The study was designed to investigate lipid profile and SYNTAX score in patients with non-ST segment elevation myocardial infarction (NSTEMI).

**Methods:**

311 patients with NSTEMI were enrolled. The demographic, clinical data, blood samples and SYNTAX score were documented. The Pearson linear correlation was used to detect confounding factors linearly correlated with SYNTAX score. The significantly correlated confounding factors were put into the multiple linear regressions.

**Results:**

The Pearson linear correlation showed that high-density lipoprotein- cholesterol (HDL-C) and apolipoprotein A1 (ApoA1) were significantly correlated with Syntax Score (*r* = − 0.119, *P* = 0.044 and *r* = − 0.182, *P* = 0.002, respectively). The multiple linear regressions for Syntax Score were built using HDL-C and ApoA1, respectively. After the adjustment of other significantly correlated confounding factors such as white blood cell count (WBC), myohemoglobin (MB), glutamic-oxalacetic transaminase (AST) and creatinine, the ApoA1 still showed significant association with Syntax Score (*β* = − 0.151, *P* = 0.028). The area under curve was (AUC) 0.624 and the optimal cutoff value is 1.07 g/L when using ApoA1 to predict moderate and severe coronary artery lesions. The patients with ApoA1 ≥ 1.07 g/L and < 1.07 g/L have the Syntax Scores of 12.21 ± 11.58 and 16.33 ± 11.53, respectively (*P* = 0.001).

**Conclusions:**

The ApoA1 is the only lipid factor significantly associated with complexity of coronary artery lesion in patients with NSTEMI, the patients with ApoA1 < 1.07 g/L may have more complex coronary artery lesions.

## Background

Non-ST segment elevation myocardial infarction (NSTEMI) is one of the most severe and lethal form of myocardial infarction [1, 2]. Although NSTEMI patients may have lower in-hospital mortality than patients with ST segment elevation myocardial infarction (STEMI), however, 4-year mortality in NSTEMI patients is two-fold higher than that in patients with STEMI [3, 4].

The SYNTAX score, a lesion-based angiographic scoring system, has been widely used to grade the complexity of coronary artery disease. Several trials have showed that patients with a relatively high SYNTAX score have worse cardiovascular outcomes, and the score is an independent predictor of major adverse cardiovascular events (MACE) for percutaneous coronary intervention (PCI) [5–7]. The SYNTAX score of ≥33 points is widely accepted criteria to select coronary artery bypass grafting (CABG) over PCI in treatment of the left main lesions. SYNTAX score of 22–32 points is defined as moderate complexity of coronary artery disease, both the CABG and PCI can be adopted [8].

Traditional lipid factors, such as total cholesterol (TC), triglycerides (TG), low-density lipoprotein cholesterol (LDL-C), high-density lipoprotein cholesterol (HDL-C) have been widely used as predictors in acute coronary syndrome [9, 10]. Apolipoprotein A1 (ApoA1), the main protein component of HDL-C in the plasma has functions of protecting against atherosclerosis and cardiovascular disease, meanwhile it could also promote cellular cholesterol efflux and cholesterol transportation from peripheral tissues to the liver [11]. Apolipoprotein B (ApoB) is the main component of LDL-C, and its level could reflect the level of atherogenic lipoprotein particles [12, 13].

A previous study has investigated the association between the lipids factors and SYNTAX score in patients with stable coronary artery disease [14]. Since vast amount of NSTEMI patients had multi-vessel disease [15, 16], the evaluation of SYNTAX score is necessary and crucial to these patients in the selection of optimal treatments. The present study intended to investigate the association between lipids factors and SYNTAX score in NSTEMI patients.

## Methods

### Subjects

We performed this present cross-sectional and observational study in Cardiology Department of First Affiliated Hospital of Xinjiang Medical University from January of 2015 to June of 2018. The NSTEMI is diagnosed according to symptoms, electrocardiogram (ECG) and myocardial enzyme results. Symptoms of NSTEMI includes the chest pain, chest tightness or dyspnea, the durations should be over 30 min. ECG diagnosis of NSTEMI is defined as: horizontal or downward ST segment depression ≥1 mm with upright, bidirectional or lightly inverted T wave, or symmetric and deep inversed T-wave without obvious ST segment shift. Myocardial enzyme changes include significant elevation of creatine kinase (CK), creatine kinase-MB (CK-MB), lactate dehydrogenase (LDH), Troponin I, myoglobin (MB), alanine aminotransferase (ALT), aspartate aminotransferase (AST), alkaline phosphatase (ALP) or transglutaminase according to features and occurrence time of each enzyme in NSTEMI patients. The patients who have symptoms, ECG and myocardial enzyme changes simultaneously are diagnosed as NSTEMI and could be enrolled in the present study. The main exclusion criteria include previous percutaneous stent implantation or percutaneous transluminal coronary angioplasty (PTCA) procedures, or previous lipid-lowering therapy and unwillingness to receive further coronary angiography (CAG) examination [17].

Three hundred eleven hospitalized NSTEMI patients were finally enrolled according to enrollment standard above, 212 (68.2%) patients were male and 99 (31.8%) patients were female. The average age was 61.80 ± 28.08 years.

### SYNTAX score

All enrolled NSTEMI patients received the CAG examination, the SYNTAX score was calculated using SYNTAX score calculator (current calculator version: 2.28; http://www.syntaxscore.com/calculator/start.htm) after the CAG procedure. Two experienced interventional cardiology physicians were in charge of calculating the SYNTAX scores. The third interventional cardiology physician should be involved in the calculation of SYNTAX scores when the scores were different. The NSTEMI patients with different SYNTAX scores were divided into two different groups, the mild lesion group (SYNTAX score < 22) and moderate and severe lesion group (SYNTAX score ≥ 22) [18].

### Clinical data and blood tests

Demographic data and cardiovascular risk factors were obtained from the medical records. The peripheral blood was sampled for myocardial enzymes tests after the admission of the patients. Other peripheral blood was sampled from patients in a fasting state on the morning following the admission day. Venous blood samples were sent to Clinical Laboratory Department of First Affiliated Hospital of Xinjiang Medical University for red blood cells (RBC) counts, hemoglobin, platelet counts, white blood cells (WBC) counts, CK, CK-MB, LDH, Troponin I, MB, ALT, AST, ALP, transglutaminase, TC, triglyceride, HDL-C, LDL-C, ApoA1, ApoB, urea nitrogen (BUN), creatinine, cystatin-C, total bilirubin (TBIL), direct bilirubin (DBIL), total protein (TP), albumin, D-Dimer (DD), glycosylated hemoglobin-A1c (HbA1c) and brain natriuretic peptide (BNP) detection using standard biochemical techniques [19].

### ECG and echocardiography

ECG was performed after the admission of the patients using Nihon Kohden ECG machine (ECG1350P). The patients with horizontal or downwardly ST segment depression ≥1 mm with upright, bidirectional or lightly inverted T wave, or symmetric and deep inversed T-wave without obvious ST segment shift were screened out for further enrollment.

The left ventricular ejection fraction (LVEF) was obtained using Doppler echocardiography conducted within 3 days of admission [20].

### Definition of risk factors

Hypertension was defined as an average systolic blood pressure ≥ 140 mmHg, or an average diastolic blood pressure ≥ 90 mmHg, or both, or self-reported use of antihypertensive medication, or a self-reported history of hypertension.

Diabetes was defined as fasting plasma glucose ≥7.0 mmol/L, or random plasma glucose ≥11.1 mmol/L, or 2 h plasma glucose in oral glucose tolerance test (OGTT) ≥ 11.1 mmol/L, or use of insulin or oral hypoglycemic agents, or a self-reported history of diabetes.

### Statistical analysis

The statistical analysis was conducted using SPSS version 16.0 for Windows (SPSS Inc., Chicago, IL, USA). Continuous variables were expressed as mean ± standard deviations and the differences between the mild lesions group and moderate and severe lesions group were analyzed using the Mann-Whitney U-test. Categorical variables were expressed as proportions and the differences in categorical variables were analyzed using chi-square test. Pearson correlation analysis was conducted to determine the correlation between SYNTAX score and each clinical and laboratory factor. The multiple linear regressions of SYNTAX score were performed using significantly correlated lipid factors, HDL-C and ApoA1, respectively. The receiver operating characteristic (ROC) curve of ApoA1 for the prediction of moderate and severe lesions was performed, then the AUC and cutoff points of ApoA1 to predict moderate and severe lesions were calculated. Additionally, the distance on the ROC curve of each ApoA1 was calculated as the square root of [(1-sensitivity)^2^ + (1 - specificity)^2^]. The ApoA1 value with the shortest distance on the ROC curve was considered as appropriate cutoff. Finally, the SYNTAX score between patients with different ApoA1 values were calculated and compared using Mann-Whitney U-test. Statistical significance was established at *P* < 0.05.

## Results

Baseline characteristics of NSTEMI patients with mild lesions or moderate and severe lesions were shown in Table 1. The percentage of male, WBC counts, troponin I, MB, BNP and SYNTAX score in NSTEMI patients with moderate and severe lesions were significantly higher than those in NSTEMI patients with mild lesions (*P* < 0.05). Meanwhile, the HDL-C, apolipoprotein A, albumin and LVEF in NSTEMI patients with moderate and severe lesions were significantly lower than those in NSTEMI patients with mild lesions (*P* < 0.05). The percentage of smoking, diabetes mellitus, hypertension and age, HR, SBP, DBP, RBC counts, hemoglobin, platelet counts, CK, CK-MB, LDH, ALT, AST, ALP, transglutaminase, TC, triglyceride, LDL-C, apolipoprotein B, BUN, creatinine, cystatin-C, TBIL, DBIL, TP, DD, HbA1c showed no significant difference between the NSTEMI patients with mild lesions or moderate and severe lesions.

**Table 1:** Baseline characteristics of NSTEMI patients with mild lesions or moderate and severe lesions

	Mild lesion (SYNTAX score < 22; *n* = 242)	Moderate and severe lesion (SYNTAX score ≥ 22; *n* = 69)	*P* value
Male (%)	154 (63.6%)	58 (84.1%)	0.001*
Smoking (%)	70 (28.9%)	25 (36.2%)	0.245
Diabetes mellitus (%)	43 (17.8%)	13 (18.8%)	0.838
Hypertension (%)	121 (50.0%)	33 (47.8%)	0.750
Age (years)	61.63 ± 31.67	62.78 ± 8.91	0.076
HR (beat/min)	73.04 ± 13.57	74.57 ± 16.06	0.477
SBP (mmHg)	129.21 ± 18.59	130.60 ± 20.39	0.796
DBP (mmHg)	78.53 ± 11.33	79.53 ± 12.14	0.843
RBC counts (× 10^12^/L)	4.54 ± 1.03	4.42 ± 0.53	0.830
Hemoglobin (g/L)	137.17 ± 16.49	138.24 ± 14.50	0.493
Platelet counts (×10^9^/L)	198.43 ± 64.01	197.26 ± 62.67	0.852
WBC counts (×10^9^/L)	7.27 ± 2.47	8.38 ± 2.82	0.005*
CK (U/L)	349.20 ± 740.63	371.30 ± 735.81	0.223
CK-MB (U/L)	33.04 ± 51.47	37.59 ± 63.13	0.322
LDH (U/L)	300.19 ± 248.24	284.07 ± 204.26	0.587
Troponin I (ng/mL)	4.03 ± 12.16	16.67 ± 100.61	0.001*
MB (ng/mL)	75.84 ± 180.60	132.54 ± 353.36	0.004*
ALT (U/L)	32.96 ± 27.62	34.70 ± 31.67	0.678
AST (U/L)	50.13 ± 70.06	66.75 ± 122.93	0.449
ALP (U/L)	95.16 ± 29.19	91.97 ± 28.21	0.756
Transglutaminase (U/L)	35.41 ± 49.02	36.68 ± 35.24	0.937
TC (mmol/L)	4.03 ± 1.06	3.92 ± 1.00	0.519
Triglyceride (mmol/L)	1.74 ± 1.52	1.62 ± 0.71	0.514
HDL-C (mmol/L)	1.08 ± 0.75	0.97 ± 0.22	0.001*
LDL-C (mmol/L)	2.21 ± 0.75	2.33 ± 0.77	0.227
Apolipoprotein A1 (g/L)	1.14 ± 0.19	1.06 ± 0.19	0.002*
Apolipoprotein B (g/L)	0.84 ± 0.22	0.88 ± 0.22	0.154
BUN (mmol/L)	5.16 ± 1.63	5.29 ± 2.16	0.898
Creatinine (umol/L)	75.75 ± 18.23	82.32 ± 26.27	0.117
Cystatin-C (mg/L)	1.50 ± 7.02	1.12 ± 0.40	0.514
TBIL (umol/L)	16.23 ± 9.97	16.52 ± 7.84	0.609
DBIL (umol/L)	5.30 ± 2.49	5.87 ± 3.24	0.163
TP (g/L)	64.16 ± 6.83	62.36 ± 5.27	0.067
Albumin (g/L)	38.75 ± 3.99	37.57 ± 3.59	0.018*
DD (ug/mL)	0.30 ± 0.58	0.33 ± 0.50	0.451
HbA1c (%)	6.25 ± 1.50	6.36 ± 1.19	0.350
BNP (pg/mL)	246.57 ± 520.58	353.81 ± 368.23	< 0.001*
LVEF (%)	58.90 ± 8.90	54.92 ± 8.83	0.001*
SYNTAX score	8.61 ± 7.09	30.87 ± 7.96	< 0.001*

Note: *NSTEMI* Non-ST-Elevation Myocardial Infarction, *HR* Heart Rate, *SBP* Systolic Blood Pressure, *DBP* Diastolic Blood Pressure, *RBC* Red Blood Cells, *WBC* White Blood Cells, *CK* Creatine Kinase, *CK-MB* Creatine Kinase-MB, *LDH* Lactate DeHydrogenase, *MB* MyogloBin, *ALT* ALanine aminoTransferase, *AST* ASpartate aminoTransferase, *ALP* ALkaline Phosphatase, *TC* Total Cholesterol, *HDL-C* High-Density Lipoprotein-Cholesterol, *LDL-C* Low-Density Lipoprotein-Cholesterol, *BUN* Urea Nitrogen, *TBIL* Total Bilirubin, *DBIL* Direct Bilirubin, *TP* Total Protein, *BNP* Brain Natriuretic Peptide, *HbA1c* Glycosylated Hemoglobin-A1c, *DD* D-Dimer, *LVEF* Left ventricular ejection fraction, *P < 0.05

Pearson correlation analysis between Syntax score and clinical and laboratory factors in NSTEMI patients was presented in Table 2. WBC counts, MB, AST and creatinine were significantly and positively correlated with SYNTAX score (*P* < 0.05). Meanwhile, HDL-C, apolipoprotein A and albumin were significantly and negatively correlated with SYNTAX score (*P* < 0.05). The age, HR, SBP, DBP, RBC counts, hemoglobin, platelet counts, CK, CK-MB, LDH, Troponin I, ALT, ALP, transglutaminase, TC, triglyceride, LDL-C, apolipoprotein B, BUN, cystatin-C, TBIL, DBIL, TP, BNP, HbA1c, DD, LVEF showed no significant correlation with SYNTAX score.

**Table 2:** Pearson correlation analysis between SYNTAX score and clinical and laboratory factors in NSTEMI patients

	*r*	*P* value
Age	0.034	0.545
HR	0.068	0.235
SBP	0.031	0.583
DBP	0.026	0.650
RBC counts	−0.018	0.750
Hemoglobin	−0.002	0.965
Platelet counts	−0.015	0.795
WBC counts	0.221	< 0.001*
CK	0.062	0.363
CK-MB	0.105	0.113
LDH	0.059	0.393
Troponin I	0.085	0.184
MB	0.155	0.015*
ALT	0.012	0.833
AST	0.184	0.001*
ALP	−0.074	0.238
Transglutaminase	0.006	0.922
TC	0.020	0.740
Triglyceride	−0.049	0.408
HDL-C	−0.119	0.044*
LDL-C	0.077	0.196
Apolipoprotein A1	−0.182	0.002*
Apolipoprotein B	0.081	0.171
BUN	−0.004	0.941
Creatinine	0.136	0.019*
Cystatin-C	0.045	0.442
TBIL	0.058	0.316
DBIL	0.084	0.147
TP	−0.094	0.103
Albumin	−0.154	0.008*
BNP	−0.021	0.744
HbA1c	0.117	0.079
DD	0.090	0.763
LVEF	−0.082	0.220

Note: *NSTEMI* Non-ST-Elevation Myocardial Infarction, *HR* Heart Rate, *SBP* Systolic Blood Pressure, *DBP* Diastolic Blood Pressure, *RBC* Red Blood Cells, *WBC* White Blood Cells, *CK* Creatine Kinase, *CK-MB* Creatine Kinase-MB, *LDH* Lactate DeHydrogenase, *MB* MyogloBin, *ALT* ALanine aminoTransferase, *AST* ASpartate aminoTransferase, *ALP* ALkaline Phosphatase, *TC* Total Cholesterol, *HDL-C* High-Density Lipoprotein-Cholesterol, *LDL-C* Low-Density Lipoprotein-Cholesterol, *BUN* Urea Nitrogen, *TBIL* Total Bilirubin, *DBIL* Direct Bilirubin, *TP* Total Protein, *BNP* Brain Natriuretic Peptide, *HbA1c* Glycosylated Hemoglobin-A1c, *DD* D-Dimer, *LVEF* Left ventricular ejection fraction, *P < 0.05

Since the significantly correlated lipid factors, HDL-C and ApoA1 were highly resembled with each other, we have built the multiple linear regression model using HDL-C and ApoA1, respectively. Multiple linear regression of SYNTAX score using ApoA1 and other confounding factors in NSTEMI patients was shown in Table 3. After the adjustment of WBC, MB, AST, albumin and creatinine, which were the confounding factors linearly correlated with SYNTAX score, the ApoA1 was still significantly associated with SYNTAX score (*β* = − 0.151, *P* = 0.028). Meanwhile, the multiple linear regression of SYNTAX score using HDL-C and other confounding factors in NSTEMI patients was shown in Table 4. After the adjustment of WBC, MB, AST, albumin and creatinine, the HDL-C was not significantly associated with SYNTAX score (*β* = 0.093, *P* = 0.167).

**Table 3:** Multiple linear regression of SYNTAX score using ApoA1 and other confounding factors in NSTEMI patients

	Unstandardized coefficients		Standardized coefficients	*P* value
	*B*	*S.E.*	*β*	
WBC	0.427	0.339	0.091	0.220
MB	0.003	0.004	0.066	0.403
AST	0.008	0.010	0.065	0.432
Albumin	− 0.181	0.199	− 0.063	0.364
Creatinine	0.056	0.041	0.093	0.174
ApoA1	−9.270	4.194	−0.151	0.028*

Note: *NSTEMI* Non-ST-Elevation Myocardial Infarction, *ApoA1* Apolipoprotein A1, *WBC* White Blood Cell, *MB* Myoglobin, *AST* Aspartate Transaminase

**Table 4:** Multiple linear regression of SYNTAX score using HDL-C and other confounding factors in NSTEMI patients

	Unstandardized coefficients		Standardized coefficients	*P* value
	*B*	*S.E.*	*β*	
WBC	0.470	0.340	0.103	0.169
MB	0.003	0.004	0.068	0.396
AST	0.009	0.010	0.073	0.385
Albumin	− 0.240	0.198	− 0.083	0.226
Creatinine	0.060	0.042	0.099	0.153
HDL-C	−4.308	3.105	0.093	0.167

Note: *NSTEMI* Non-ST-Elevation Myocardial Infarction, *HDL-C* High Density Lipoprotein Cholesterol, *WBC* White Blood Cell, *MB* Myoglobin, *AST* Aspartate Transaminase

**Fig. 1:**
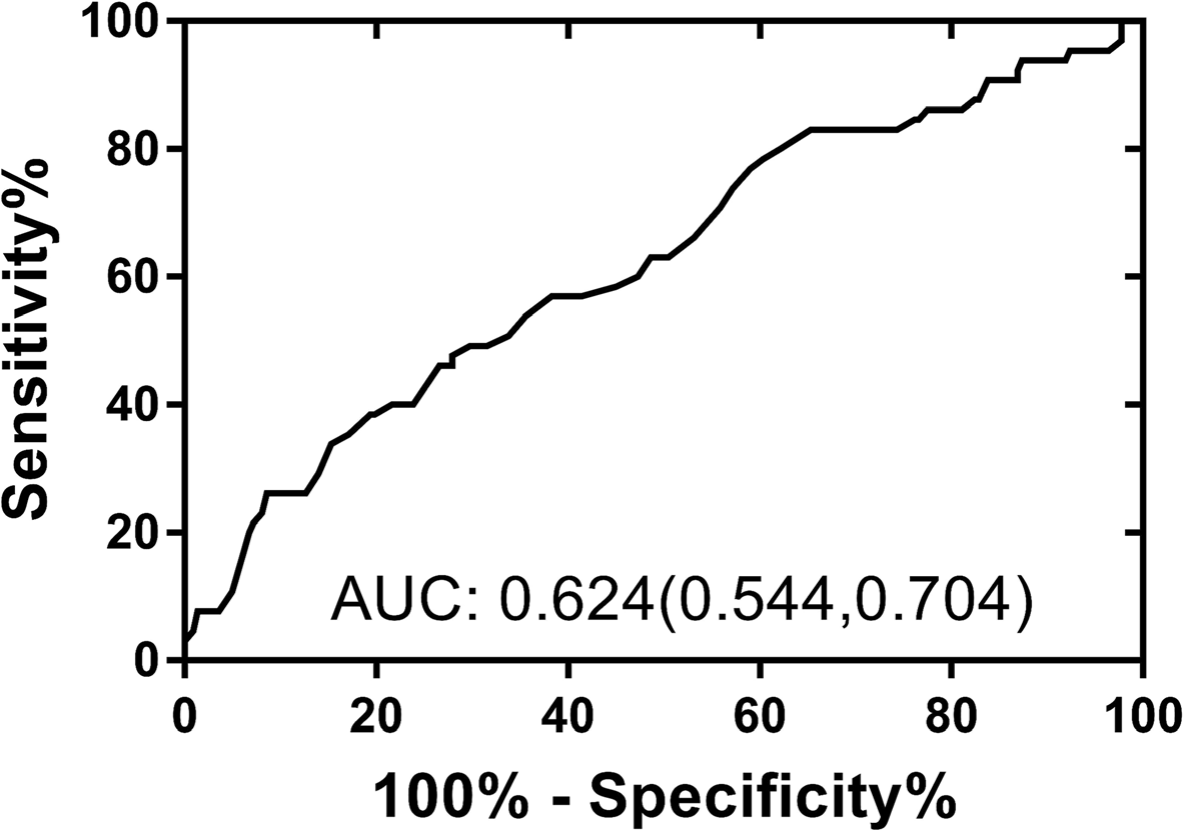
The ROC curve of ApoA1 for the prediction of moderate and severe lesions in NSTEMI patients

The ROC curve of ApoA1 for the prediction of moderate and severe lesions in NSTEMI patients was presented in Fig. 1. The AUC of ApoA1 for the prediction of moderate and severe lesions was 0.624 (0.544–0.704). The cutoff points of ApoA1 to predict moderate and severe lesions were calculated in Table 5. The distances in ROC curve were calculated using various ApoA1 values, the shortest distance on the ROC curve was 0.579 and 1.07 g/L of ApoA1 was considered as the optimal cutoff in the prediction of moderate and severe lesions in NSTEMI patients with the sensitivity of 0.631 and specificity of 0.554.

**Table 5:** Sensitivity, specificity, and distance in the ROC curve of ApoA1 for the prediction of moderate and severe coronary artery lesions in NSTEMI patients

ApoA1 cutoffs (g/L)	Sensitivity	Specificity	Distance in ROC curve
0.75	1.000	0.000	1.000
0.80	0.996	0.039	0.961
0.90	0.910	0.262	0.744
1.00	0.748	0.431	0.622
1.06	0.653	0.523	0.590
1.07	0.631	0.554	0.579
1.08	0.602	0.569	0.587
1.09	0.568	0.577	0.605
1.10	0.539	0.593	0.615
1.11	0.521	0.616	0.614
1.20	0.334	0.831	0.687
1.30	0.200	0.862	0.812
1.40	0.093	0.938	0.910
1.50	0.041	0.954	0.960
1.85	0.000	1.000	1.000

Note: *NSTEMI* Non-ST-Elevation Myocardial Infarction, *ROC* Receiver operating characteristic curve, *ApoA1* Apolipoprotein A1

**Fig. 2:**
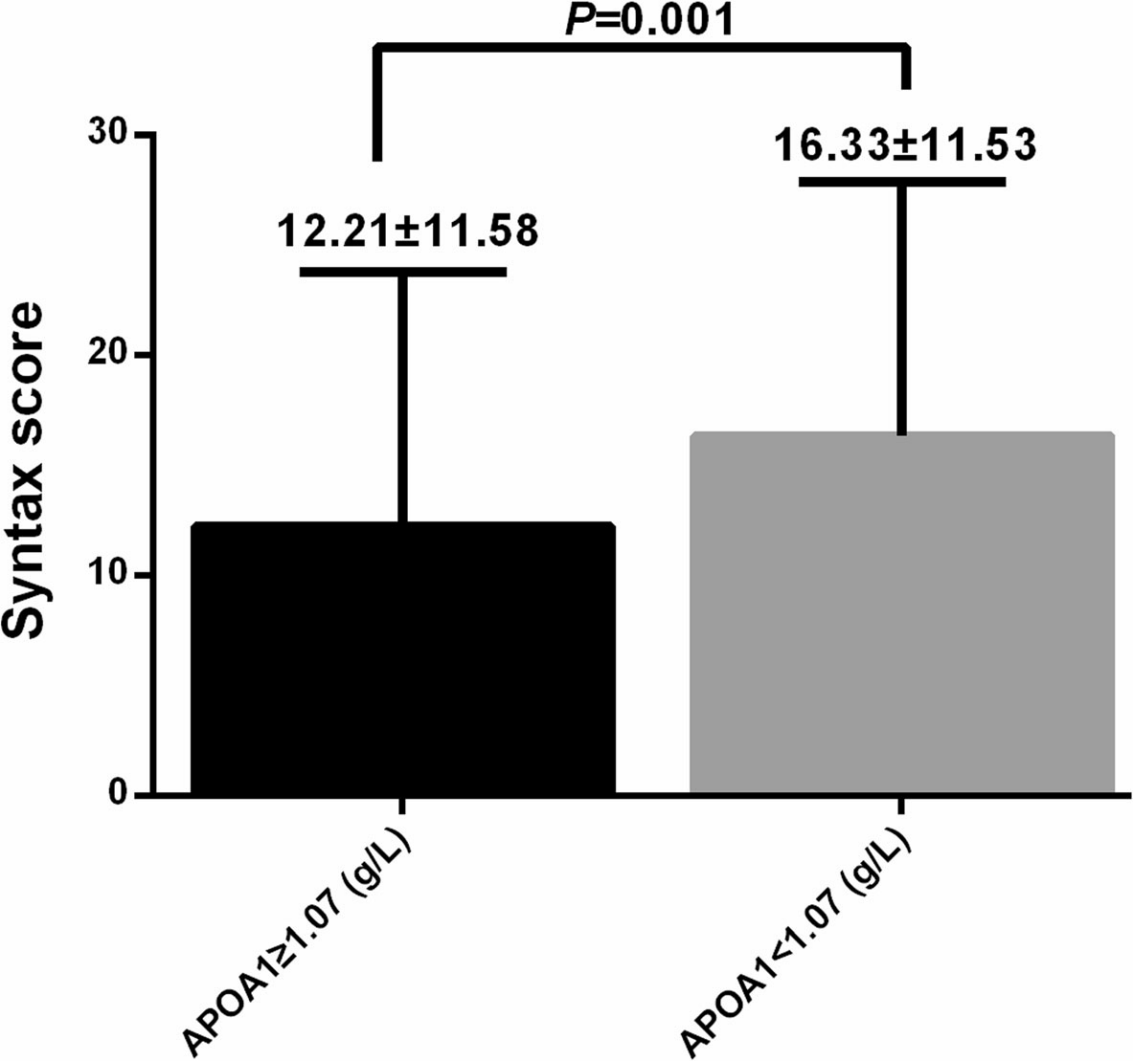
The SYNTAX score between patients with ApoA1 ≥ 1.07 and ApoA1 < 1.07

The SYNTAX score between patients with ApoA1 ≥ 1.07 and ApoA1 < 1.07 were presented in Fig. 2. The patients with ApoA1 < 1.07 had significantly higher SYNTAX score (16.33 ± 11.53 g/L) than the patients with ApoA1 ≥ 1.07 (12.21 ± 11.58 g/L).

## Discussion

Previous studies have reported that up to 80% of NSTEMI patient had multi-vessel disease, the complexity of coronary artery disease in NSTEMI patients was much higher than those in STEMI patients [16, 21]. The appliance of the SYNTAX score was crucial since it reflected the complexity of coronary artery lesions in NSTEMI patients with more complicated coronary artery lesions. Meanwhile, Garg et al. [22] demonstrated that the SYNTAX score has the power to predict major adverse cardiovascular events and mortality. Other studies have also reported that the adverse long-term cardiovascular events showed significant increase as the SYNTAX score increased [23–25]. The SYNTAX score is an effective tool to affect the decision-making between CABG and PCI [26]. If the SYNTAX score was ≥33 points in the left main lesion, the CABG should be considered as the priority treatment rather than PCI. In this present study, 242 patients (77.8%) had SYNTAX score < 22 points and 69 patients (22.2%) had SYNTAX score ≥ 22 points, the patients with moderate and severe lesions had mean ± S.D. SYNTAX score of 30.87 ± 7.96 points.

Among the documented lipid factors, the present study showed that HDL-C and ApoA1 were linearly and significantly correlated with SYNTAX score. Since the HDL-C and ApoA1 were highly resembled with each other, we have built the multiple linear regression models using HDL-C and ApoA1, respectively. After the adjustment of WBC, MB, AST, albumin and creatinine, the ApoA1 was still significantly associated with SYNTAX score (*β* = − 0.151, *P* = 0.028). However, after the adjustment of WBC, MB, AST, albumin and creatinine, the HDL-C was not significantly associated with SYNTAX score (*β* = 0.093, *P* = 0.167). The mechanism of the association between the ApoA1 and SYNTAX score in NSTEMI patients is not entirely clear. The possible mechanism may be as follows, human or recombinant ApoA1 has been shown to increase HDL-C efflux capacity and to reduce atherosclerotic disease. Several studies have identified ApoA1 as a key determinant of macrophage cholesterol efflux capacity, ApoA1 proteins may reduce atherosclerosis via regulating cholesterol efflux from macrophages [9, 27]. Another study has made a reconstituted infusible human ApoA1, CSL112, the CSL112 were injected to ACS patients and patients with stable atherosclerotic disease, the function of acute cholesterol efflux enhancement was confirmed in both population, and the benefit of CSL112 to reduce major adverse cardiovascular events remained to be seen in the following study [11, 28].

The study also demonstrated that The AUC of ApoA1 for the prediction of moderate and severe lesions was 0.624 (0.544–0.704) and 1.07 g/L of ApoA1 was considered as the optimal cutoff in the prediction of moderate and severe lesions in NSTEMI patients with the sensitivity of 0.631 and specificity of 0.554. The patients with ApoA1 < 1.07 had significantly higher SYNTAX score (16.33 ± 11.53 g/L) than the patients with ApoA1 ≥ 1.07 (12.21 ± 11.58 g/L). The 1.07 g/L of ApoA1 was selected as the optimal cutoff in the prediction of moderate and severe lesions, the NSTEMI patients with ApoA1 < 1.07 g/L may have more severe coronary artery lesions. The establishment the cutoff values added the clinical use to possible coronary artery lesions, the physicians should be alerted when the ApoA1 was < 1.07 g/L since multi-vessel or severe coronary artery lesions may lead to worse clinical outcomes.

Aksakal et al. [29] reported that the presence of diabetes mellitus, no previous statin use, lower values of HDL-C, LVEF, estimated glomerular filtration rate were independent predictors of coronary artery complexity assessed using the SYNTAX score. This study has confirmed the HDL-C and creatinine were linearly correlated with coronary artery complexity. Meanwhile, the LVEF was significantly different between the patients with mild lesion and patients with moderate and severe lesion. However, the presence of diabetes mellitus and HbA1c showed no relationship with the SYNTAX score, the difference may come from the enrollment of patients, our patients were NSTEMI patients while the majority of patients from Aksakal et al. [29] were stable coronary artery disease patients. The study also documented the MB and AST were linearly correlated with SYNTAX score, the level of MB and AST may reflect the myocardial injury in NSTEMI patients, the level of myocardial injury may reflect the complexity and severity of coronary artery lesions determined by SYNTAX score [30].

The strengths of the study are that it is the first association study between the lipid factors and complexity of coronary artery lesions in NSTEMI patients. Second, the study enrolled the cardiac enzymes and myocardial damage marker such as CK, CK-MB, LDH, Troponin I, MB, ALT, AST, ALP and transglutaminase, the significant different markers were adjusted as confounding factors. Third, the study excluded previous percutaneous stent implantation or percutaneous transluminal coronary angioplasty (PTCA) procedures, the SYNTAX score may precisely reflect the original status in NSTEMI patients, the study also excluded the patients with previous lipid-lowering therapy, which guaranteed the accuracy of original lipids status. The study has several limitations, it is an cross-section study that it could not determine the causal relationship between the ApoA1 and SYNTAX score in NSTEMI patients. Second, the significantly different distributed gender was not in the linear regression since it is a categorical, the limited women number made it difficult for subgroup analysis. Third, we have observed that the *P* value for HDL-C was 0.167, meanwhile, the LDL-C and Apo B were also not correlated with SYNTAX score, the significance may change if the more NSTEMI patients were enrolled.

## Conclusion

In conclusion, the ApoA1 is associated with SYNTAX score in NSTEMI patients. 1.07 g/L of ApoA1 was considered as the optimal cutoff in the prediction of moderate and severe lesions in NSTEMI patients with the sensitivity of 0.631 and specificity of 0.554. The further study may need larger sample size to further detect the association HDL-C, LDL-C, Apo B and SYNTAX score in NSTEMI patients, meanwhile, the underlying mechanisms in both animal and human models, and the effectiveness and safety of supplementary therapy in human still needed further exploration.

## Data Availability

Data and material were available.

## Abbreviations

ALP: alkaline phosphatase;
ALT: alanine aminotransferase
ApoA1: apolipoprotein A1
ApoB: Apolipoprotein B
AST: aspartate aminotransferase
AUC: area under curve
BNP: brain natriuretic peptide;
BUN: urea nitrogen;
CABG: coronary artery bypass grafting
CAG: coronary angiography
CK: creatine kinase
CK-MB: creatine kinase-MB
DBIL: direct bilirubin
DD: D-Dimer
ECG: electrocardiogram
HbA1c: glycosylated hemoglobin-A1c
HDL-C: high-density lipoprotein- cholesterol
LDH: lactate dehydrogenase
LDL-C: low-density lipoprotein cholesterol
LVEF: left ventricular ejection fraction
MACE: major adverse cardiovascular event
MB: myoglobin
NSTEMI: non-ST segment elevation myocardial infarction
OGTT: oral glucose tolerance test;
PCI: percutaneous coronary intervention
PTCA: percutaneous transluminal coronary angioplasty
RBC: red blood cells
ROC: receiver operating characteristic
STEMI: ST segment elevation myocardial infarction
TBIL: total bilirubin
TC: cholesterol
TG: triglycerides
TP: total protein
WBC: white blood cell count
